# Burden of Mendelian disorders in a large Middle Eastern biobank

**DOI:** 10.1186/s13073-024-01307-6

**Published:** 2024-04-08

**Authors:** Waleed Aamer, Aljazi Al-Maraghi, Najeeb Syed, Geethanjali Devadoss Gandhi, Elbay Aliyev, Alya A. Al-Kurbi, Omayma Al-Saei, Muhammad Kohailan, Navaneethakrishnan Krishnamoorthy, Sasirekha Palaniswamy, Khulod Al-Malki, Saleha Abbasi, Nourhen Agrebi, Fatemeh Abbaszadeh, Ammira S. Al-Shabeeb Akil, Ramin Badii, Tawfeg Ben-Omran, Bernice Lo, The Qatar Genome Program Research Consortium, Said I. Ismail, Wadha Al-Muftah, Radja Badji, Hamdi Mbarek, Dima Darwish, Tasnim Fadl, Heba Yasin, Maryem Ennaifar, Rania Abdellatif, Fatima Alkuwari, Muhammad Alvi, Yasser Al-Sarraj, Chadi Saad, Asmaa Althani, Eleni Fethnou, Fatima Qafoud, Eiman Alkhayat, Nahla Afifi, Sara Tomei, Wei Liu, Kun Wang, Stephan Lorenz, Hakeem Almabrazi, Fazulur Rehaman Vempalli, Ramzi Temanni, Tariq Abu Saqri, Mohammedhusen Khatib, Mehshad Hamza, Tariq Abu Zaid, Ahmed El Khouly, Tushar Pathare, Shafeeq Poolat, Rashid Al-Ali, Omar Albagha, Souhaila Al-Khodor, Mashael Alshafai, Lotfi Chouchane, Xavier Estivill, Hamdi Mbarek, Jithesh V. Puthen, Karsten Suhre, Zohreh Tatari, Younes Mokrab, Khalid A. Fakhro

**Affiliations:** 1 Department of Human Genetics, Sidra Medicine, Doha, Qatar; 2 Applied Bioinformatics Core, Sidra Medicine, Doha, Qatar; 3 Diagnostic Genomic Division, Hamad Medical Corporation, Doha, Qatar; 4 Section of Clinical and Metabolic Genetics, Department of pediatrics, Hamad Medical Corporation, Doha, Qatar; 5 Department of Pediatric, Weill Cornell Medical College, Doha, Qatar; 6 Division of Genetic & Genomics Medicine, Sidra Medicine, Doha, Qatar; 7 College of Health and Life Sciences, Hamad Bin Khalifa University, Doha, Qatar; 8 Department of Genetic Medicine, Weill Cornell Medicine-Qatar, Doha, Qatar; 9 College of Health Sciences, Qatar University, Doha, Qatar

**Keywords:** Mendelian disorders, Rare genetic disease, Genome sequencing, Consanguinity, Qatar, Middle East, Biobank, Arab population, Pathogenic variants

## Abstract

**Background:**

Genome sequencing of large biobanks from under-represented ancestries provides a valuable resource for the interrogation of Mendelian disease burden at world population level, complementing small-scale familial studies.

**Methods:**

Here, we interrogate 6045 whole genomes from Qatar—a Middle Eastern population with high consanguinity and understudied mutational burden—enrolled at the national Biobank and phenotyped for 58 clinically-relevant quantitative traits. We examine a curated set of 2648 Mendelian genes from 20 panels, annotating known and novel pathogenic variants and assessing their penetrance and impact on the measured traits.

**Results:**

We find that 62.5% of participants are carriers of at least 1 known pathogenic variant relating to recessive conditions, with homozygosity observed in 1 in 150 subjects (0.6%) for which Peninsular Arabs are particularly enriched versus other ancestries (5.8-fold). On average, 52.3 loss-of-function variants were found per genome, 6.5 of which affect a known Mendelian gene. Several variants annotated in ClinVar/HGMD as pathogenic appeared at intermediate frequencies in this cohort (1–3%), highlighting Arab founder effect, while others have exceedingly high frequencies (> 5%) prompting reconsideration as benign. Furthermore, cumulative gene burden analysis revealed 56 genes having gene carrier frequency > 1/50, including 5 ACMG Tier 3 panel genes which would be candidates for adding to newborn screening in the country. Additionally, leveraging 58 biobank traits, we systematically assess the impact of novel/rare variants on phenotypes and discover 39 candidate large-effect variants associating with extreme quantitative traits. Furthermore, through rare variant burden testing, we discover 13 genes with high mutational load, including 5 with impact on traits relevant to disease conditions, including metabolic disorder and type 2 diabetes, consistent with the high prevalence of these conditions in the region.

**Conclusions:**

This study on the first phase of the growing Qatar Genome Program cohort provides a comprehensive resource from a Middle Eastern population to understand the global mutational burden in Mendelian genes and their impact on traits in seemingly healthy individuals in high consanguinity settings.

**Supplementary Information:**

The online version contains supplementary material available at 10.1186/s13073-024-01307-6.

## Background

Having an aggregate prevalence of up to 1 in 50 children, Mendelian disorders often cause profound socioeconomic impact [1] with many unsolved cases embarking on lengthy diagnostic odyssey [2]. Anticipating and diagnosing Mendelian disorders early and at scale are high priorities for healthcare providers worldwide, many of which started adopting high throughput technologies at the point-of-care, notably next-generation sequencing (NGS) [3, 4]. To date, over 7000 monogenic human disorders have been reported, caused by genetic variants in over 4000 genes [5, 6]. While most of these disorders are presently incurable, knowledge of their molecular etiologies would lead to better patient management [7], whether through earlier and more accurate genetic screening (including pre-marital, preimplantation, and neonatal) or enhanced/new treatment [8–10].

Despite the increasing use of NGS technologies, the diagnostic yield of Mendelian disorders remains low, partially due to the rarity of these conditions and the difficulty of identifying causal pathogenic variants from the large numbers of other private variants detectable in an individual’s genome and interpreting their functional impact. Traditionally, family-based segregation analysis has been useful in identifying pathogenic variants; nevertheless, it requires genomic annotation at population level which increasingly made available from global genomic consortia on rare diseases as well as unaffected subjects [5, 6, 11–14]. Despite the success of these global efforts, they remain limited in terms of ancestral representation from non-European populations such as Middle Eastern, South Asian, and African [15, 16].

The wide Middle Eastern region (North Africa, Levant, Arabian Peninsula and Western Asia) is known for high consanguinity (in Qatar estimated at 35–54% [17, 18]), large family size, and high incidence of recessive diseases [19], especially congenital and metabolic disorders [20]. Notably, there is clustering of Mendelianized forms of complex disease in various tribes/subpopulations, reflecting founder effect [3, 21]. Recently, the Qatari population has been shown to be genetically diverse, consisting of five main genetic ancestries: Peninsular Arabs (PAR), General Arabs (GAR), West Eurasian and Persian Arabs (WEP), South Asian Arabs (SAS), and African Arabs (AFR) [16, 22], each characterized with different demographic histories and patterns of consanguinity and disease risks [3, 23]. To date, most studies on these ancestries have either been limited in size [24], scope [25, 26], or family-based. Previous studies that looked at the burden of disease in population biobanks focus mainly on populations of European ancestry [27] or regions of the world where consanguinity is low and risk of founder variants is minimal.

**Fig. 1:**
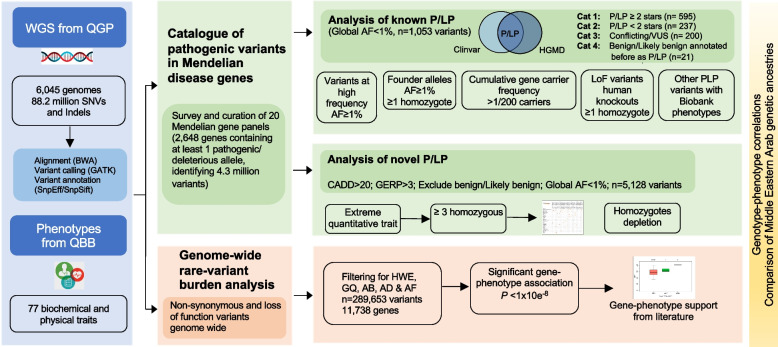
Summary of study on the burden of Mendelian disorders in a large Middle Eastern biobank from Qatar. An in-depth analysis of pathogenic variants for Mendelian disorders among 6045 Qatari genomes with 77 biochemical and clinical phenotypes obtained from Qatar biobank. The study consists of two parts: first, investigation of the landscape of known and novel pathogenic variants; second, genome wide rare variant burden analysis. Pathogenic and likely pathogenic variants (P/LP) were defined based on intersecting ClinVar and HGMD. Global AF indicates allele Frequency in international databases including 1000 genomes, gnomAD, and ExAC. Other acronyms are explained as follows: AB, allele balance, AD, allele depth; AF, allele frequency

Here, we present the first large-scale systematic analysis of Mendelian disease burden in Qatar, a proxy population for the Arabian Peninsula [22] (Fig. 1). We combine genome sequencing (WGS) data and clinically relevant phenotypic traits for 6045 healthy volunteers from the Qatar Biobank (QBB) released as part of Qatar Genome Program (QGP) [28]. Building on recent work on actionable variants among secondary findings (SF) relating to genes listed by the American College of Medical Genetics and Genomics (ACMG) [29], we map the broad landscape of known pathogenic variants across a multitude of Mendelian disease gene panels. Also, we annotate novel candidate functional variants in these genes, constituting a comprehensive catalog of known and putative pathogenic variants covering the main Middle Eastern Arab ancestries. Furthermore, we run genome-wide rare-variant burden analysis against various quantitative traits to identify novel gene-phenotype associations. By providing new insight about Mendelian disease epidemiology in Qatar and the Middle East, the results from this study will be valuable in supporting genomic medicine in the region and worldwide.

## Methods

### Sample collection

The study cohort consists of 6218 subjects from the Qatar Biobank project [30, 31], a national project that collects phenotype data and biological samples from the local population of Qatar. WGS data for this cohort was generated at Sidra Medicine as part of phase 1 of the Qatar Genome Program (QGP [28]). We used phenotypic data collected by Qatar Biobank on self-reported health status and disease history as well as 58 quantitative traits including anthropometric descriptors, hematological, and biochemical biomarkers that were measured on participants/their samples by medical personnel at QBB [30, 31] (Additional file 1: Table S1). All participants were consented for the study. Approvals were obtained from the institutional review boards at Sidra Medicine and Qatar Biobank (Ex/2017/QGP-RES-PUB-003-0011).

### Genome sequencing and data processing

Genome sequencing data was generated as described previously [22]. All samples were sequenced to a minimum average depth of 30x and processed using standard pipelines [28]. In brief, the raw sequencing data (Fastq) were first subject to quality checking using FastQC, v0.11.2. The sequencing reads were then mapped to the reference genome (hs37d5) using BWAkit v0.7.1229, and variants were called using GATK, following their recommended best practices [32]. Variants with “PASS” Quality filter were retained for downstream analysis. The VCF files were annotated using SnpEff/SnpSift [33] including information from dbSNP build-151 [34], ClinVar 2019-02-11 [35], HGMD [36], allele frequencies from gnomAD [37], 1000 genomes project [38], Greater Middle East genome project [19], and the GenomeAsia100K [39]. Quality control at the sample level revealed 173 subjects had gender mismatch, excess of heterozygosity, duplication, low call rate, or outlier positions in PCA analysis. The final number of samples used in downstream analysis was 6045. Details of this QC are described in [40].

### Gene panels

An extensive list of genes linked to a broad spectrum of human Mendelian disorders were manually curated from various sources (Additional file 1: Table S2). First, 3891 genes were retrieved for 16 panels form Genomic England PanelApp [41], retaining only genes with a source classification of “Expert_Review_Green” (which indicates reviewed by at least three independent sources). Moreover, we added 382 genes found from two other panels reported to cause severe recessive disorders and/or to lead to embryonic or neonatal lethality [42, 43]. Also, we added the latest version of ACMG Secondary Finding genes (*n* = 73) (ACMG-SF v3.1) [44]. To that, 187 genes not part of any known gene panels but with reported pathogenic/likely pathogenic variants in ClinVar were added as a separate panel labeled “Other.” In total, the obtained Mendelian disease gene list contained 2648 unique genes across 20 panels. These were finally annotated with reported patterns of inheritance using the Online Mendelian Inheritance in Man (OMIM) database [OMIM [22]].

### Genetic ancestry assignments

Study subjects were assigned into their corresponding Arab genetic ancestry groups as per our recent study [22]. These groups are General Arabs (GAR), Peninsular Arabs (PAR), West Eurasians and Persian Arabs (WEP), African Arabs (AFR), South Asian Arabs (SAS), and an Admixed Arab (ADM) group.

### Identification of known and novel pathogenic variants

Known disease-causing variants were identified in all genes as follows. First, we selected variants located in genes reported in ClinVar or HGMD with maximum allele frequency < 1% in any external global population. From those, we retained variants affecting the coding region (i.e., missense, nonsense, frameshift, and splice-site variants) and non-coding region flagged as “Pathogenic/Likely Pathogenic” in ClinVar and disease-causing “DM/DM?” in HGMD. These variants were classified into 4 categories based on the latest ClinVar variant status (as of June 2023): category 1 (P/LP ≥ 2 stars), category 2 (P/LP < 2 stars), category 3 (Conflicting/VUS), and category 4 (Benign/Likely benign annotated before as P/LP). Throughout the text, we generally refer to P/LP the variants in these categories.

Novel (putative) pathogenic variants were identified in the curated list of Mendelian genes as follows. We selected variants with minor allele frequency (AF) < 1% in external global databases, with CADD > 20, GERP > 3, having at least 3 homozygotes, and excluding benign and likely benign variants. Homozygotes of these novel alleles were examined for extreme quantitative values across the 58 quantitative traits and clinical records obtained from QBB.

### Cumulative gene carrier frequency

Cumulative gene carrier frequencies (GCFs) were estimated for known P/LP variants per subpopulation as described previously [45]. In brief, variant carrier frequency was calculated as AC – Hom/(0.5xAN), where AC is allele count and AN is the total number of alleles in unrelated individuals for a given QGP subpopulation. GCFs were calculated by summing the variant carrier frequencies per gene. Genes with GCF > 1/200 carriers were selected as meeting the criteria for being listed as ACMG Tier 3 genes and further classified into 4 categories: category 1 (> 1/50), category 2 (> 1/100–1/50), category 3 (> 1/150–1/100), and category 4 (> 1/200–1/150) as performed elsewhere [46].

### Rare variant burden analysis

Burden analysis was performed per gene including rare variants (AF < 1% in the study cohort and various external databases) that passed stringent QC criteria including allelic balance of heterozygote > 0.2 or < 0.8 and homozygote > 0.80, genotype quality > 10, call rate > 90%, Hardy-Weinberg equilibrium *P* < 1 × 10^−6^, excluding doubletons with allele depth ≥ 10 and singletons. This involved applying linear-mixed model against each quantitative trait, adjusting for age, gender, and the first four principal components of PCA, as implemented in Hail (https://hail.is).

## Results

### Study cohort characteristics

We examined 6045 volunteers enrolled at the Qatar Biobank (QBB), having WGS data [28], 58 anthropometric measurements/clinical biochemistry traits (Additional file 1: Table S1), and questionnaires on family disease history and various socio-economic parameters [30, 31]. As indicated in Table 1, the average age of subjects is 40 years with 1.28 male to female ratio, reported parental consanguinity is 29.1%, and the participants belong to 6 genetic groups as previously described [22] (QGP-GAR, QGP-WEP, QGP-PAR, QGP-AFR, QGP-SAS, and QGP-ADM) with the first three groups comprising 78.3% of subjects.

**Table 1:** Characteristics of the study cohort

**Demographic characteristic**	**Value**
Number of participants	6045
Gender (male:female)	3402:2643
Mean age (Q1–Q2)	40 (30–49)
Parent consanguinity^a^	29.1%
**Number of participants per Sub-population ancestry**^**b**^	
General Arabs (QGP-GAR)	2311 (38.2 %)
West-Asian Persian Arabs (QGP-WEP)	1372 (22.7%)
Admixed Arabs (QGP-ADM)	1180 (19.5 %)
Peninsular Arabs (QGP-PAR)	1052 (17.4 %)
African Arabs (QGP-AFR)	92 (1.5 %)
South Asian Arabs (QGP-SAS)	38 (0.6 %)

^a^Self-reported parent consanguinity (1st or 2nd degree cousins)

^b^Genetic ancestries were adapted from Razali et al. 2021

### Generating a catalog of Mendelian disease genes and variants in the Qatari population

**Fig. 2:**
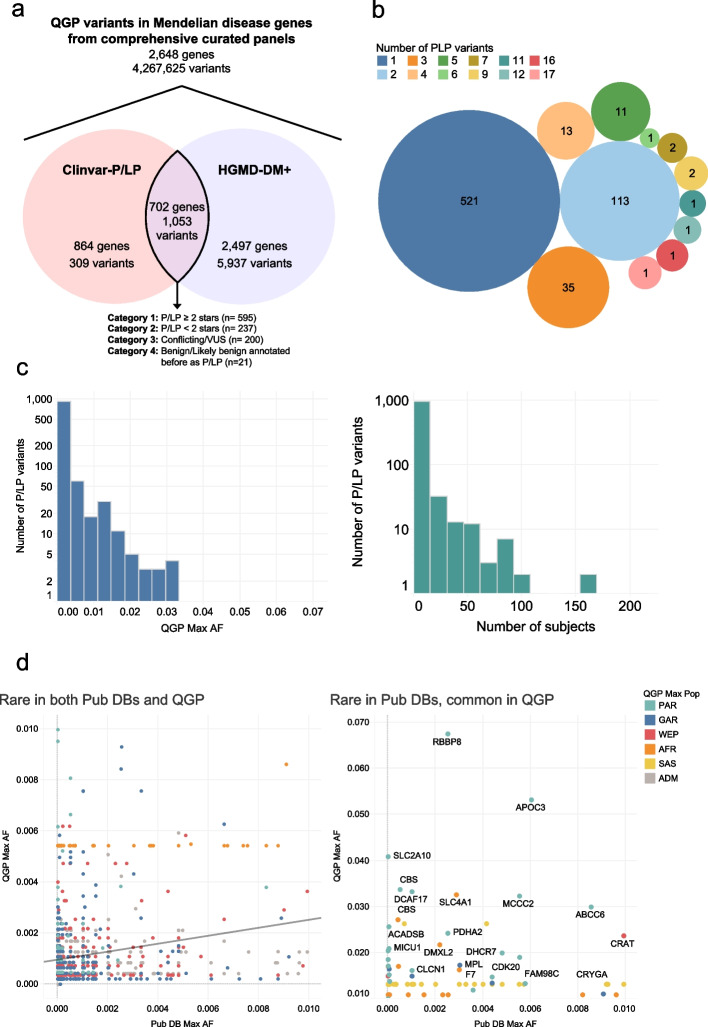
Identification and description of pathogenic variants in 6,045 Qatari genomes. **a** Known pathogenic and likely pathogenic variants (P/LP) were obtained by intersecting variants from the Qatari cohort with 20 curated gene panels, ClinVar and HGMD databases. P/LP were defined as those with classes of “Pathogenic” and/or “Likely pathogenic” in ClinVar and “DM” or “DM?” categories in HGMD. Abbreviations; Cln-PLP, ClinVar pathogenic/likely pathogenic; HGMD-DM+, HGMD class of “DM” and/or “DM?”. **b** Count of genes per number of P/LP variants. **c** Allele frequency distribution of P/LP variants indicating most are below 1% (Left). Count of P/LP variants versus number of carrier subjects (Right). **d** Correlation of allele frequencies of P/PL variants in the Qatari dataset and global databases for variants that are rare in both cohorts (Left) and for those that are common in Qatari dataset and rare in global databases (Right)

A comprehensive survey and curation of Mendelian gene panels from various literature sources was conducted (“ Methods”), resulting in a final set of 2648 unique Mendelian genes across 20 panels, associated with 2116 diseases/phenotypes (including 1261 recessive and 561 dominant diseases) (Additional file 1: Table S2). From the total number of variants in the study cohort (74,991,446 single-nucleotide variants (SNVs) and 13,199,792 insertion-deletions (Indels)), 4.8% (4,265,480) overlapped with these genes and were rare (AF < 1%) in public databases. To identify known pathogenic and likely pathogenic variants from these variants, we selected those annotated as “Pathogenic” or “Likely_pathogenic” in ClinVar and at the same time as “DM” or “DM?” in human gene mutation database (HGMD), leveraging the independent annotation of these two databases. This resulted in 1053 variants (927 SNVs and 126 indels), affecting 702 unique genes in our panels (Additional file 1: Table S3). Next, we used the latest ClinVar variant status (as of June 2023) to classify these variants into four categories and denote variants in categories 1 and 2 as P/LP (described in the “ Methods” section, Fig. 2a).

### Spectrum of known P/LP variation in the Qatari population

The QBB cohort was aimed to enroll generally healthy adults. Consistently, 40 subjects from the study cohort (0.6%) were homozygous for at least one recessive P/LP genotype, whereas 233 subjects (3.9%) were heterozygous for P/LP variants for dominant conditions with variable penetrance. Overall, 3870 subjects from the cohort (64.0%) carried at least one allele of a P/LP variant from the Mendelian panels, and 2417 subjects (62.5%) carried at least 1 P/LP allele in genes relating to autosomal recessive conditions (ARCs), reflecting the history of consanguinity in the population. On average, we found 1.1 P/LP variants per genome (range 0-7) (Additional file 2: Fig. S1, Table 2).

**Table 2:** Distribution of P/LP variants per Qatar subpopulation

**Subpopulation**	**Carriers of P/LP variants (%)**	**Average P/LP variants per subject (range)**	**Homozygotes of P/LP variants for recessive conditions (%)**	**Fold enrichment of homozygote P/LP relative to ADM**	**Heterozygotes of P/LP variants in dominant conditions (%)**	**Fold enrichment of heterozygote P/LP relative to ADM**
QGP-PAR	794 (75)	1.4 (0–6)	25 (3.15)	5.8^a^	22 (2.8)	0.4
QGP-GAR	1507 (65)	1.1 (0–7)	10 (0.66)	1.2	71 (4.7)	0.6
QGP-ADM	739 (63)	1.0 (0–6)	4 (0.54)	1	55 (7.4)	1
QGP-AFR	60 (65)	1.0 (0–7)	0 (0)	0	8 (13.3)	1.8
QGP-SAS	19 (50)	0.7 (0–3)	0 (0)	0	1 (5.3)	0.7
QGP-WEP	747 (54)	0.8 (0–5)	1 (0.13)	0.2	76 (10.1)	1.4

^a^Significant enrichment (Wilcoxon rank sum test)

In terms of distribution of P/LP variants across genes, 696 genes (99.1%) had less than five P/LP variants, while 75.2% had only one variant (Fig. 2b). As expected, most of the P/LP variants (*n* = 984) were rare across the various Qatari subpopulations (AF < 1%) (Fig. 2c), with only 31 affected actionable secondary finding genes (ACMG-SF) (Additional file 1: Table S4); 29 of which were reported earlier [29].

**Fig. 3:**
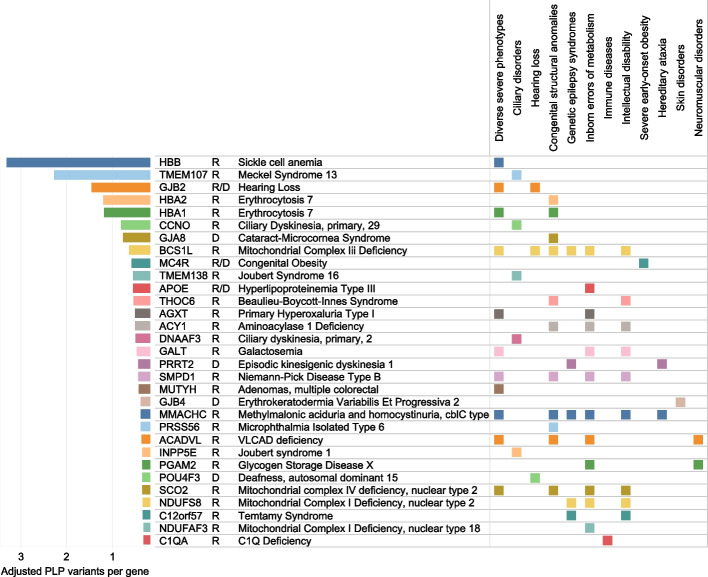
Genes with the most pathogenic alleles and corresponding disease phenotypes and panels. Number of P/LP variants was calculated per gene and adjusted for gene length, showing genes with adjusted counts > 0.3. OMIM phenotype and inheritance mode are shown for each gene, in addition to gene panel membership

Relevant to population public health, we investigated burden per gene panels. We observed the highest P/LP burden in genes related to severe/recessive lethal conditions, ciliary disorders, hearing loss, and congenital structural anomalies, with 5 genes harboring more than 1 P/LP variants per gene adjusted for gene length: *HBB*, *TMEM107*, *GJB*2, *HBA2*, and *HBA1*) (Fig. 3).

In terms of ancestry-level distribution by zygosity, we found that Peninsular Arab subpopulation has the least enrichment for heterozygous carriers of P/LP variants relative to other ancestral subpopulations; however, for homozygous P/LP variants, it has significantly higher enrichment (~5.8 fold higher than the admixed group) (Wilcoxon-rank sum test, *P=* 2.1e−6) (Table 2).

### P/LP variants exceedingly common in the Qatar population

We found 18 P/LP variants have maximum AF ≥ 2% in the Qatari subpopulation, 12 of which had homozygous carriers ranging from 1 to 10 (Additional file 1: Table S5). Three of these variants had maximum AF > 4% (found in the PAR subpopulation): (1) a missense variant (rs373804633; category 3; c.298C>T; p.Arg100Trp) in *RBBP8*, a gene associated with Seckel syndrome (MIM# 606744), (2) loss-of-function variant in *APOC3* (rs76353203; category 3; c.109C>T: p.Arg37* associated with apolipoprotein C-III deficiency (MIM# 614028) [47] that characterized by low levels of triglycerides (discussed in details below), and (3) missense variant in *SLC2A10* (rs80358230; category 2; c.243C>G; p.Ser81Arg) which is associated with arterial tortuosity syndrome (MIM# 208050) [48]. While carriers of the *APOC3* variant have consistently lower levels of triglycerides, carriers of the variants in the other 2 genes do not seem to have traits in the biobank pointing to the associated conditions, despite their severity.

### Founder P/LP alleles in the Qatari population

To identify putative founder P/LP variants in each of the five QGP subpopulations, subpopulation-level allele frequencies were compared against global databases. The correlation of rare variants (AF < 1%) among QGP subpopulations as well as global datasets shows relatively low correlation (Pearson’s correlation, *r*^2^ = 0.22), highlighting population-specific distribution of known pathogenic variants (Fig. 2d, left).

By looking at known P/LP variants with AF > 1% in the Qatari subpopulations and with AF < 1% in global datasets, 59 variants were identified as potential founder alleles including 23 that are either absent or have extremely low frequency in public databases (Additional file 1: Table S5; Fig. 2d, right). The highest differences in terms of allele frequency is observed in the PAR subpopulation, where previously known founder variants have also been observed, including the variant in *DCAF17* (rs797045038; category 1; c.436delC; p.Ala147fs), associated with Woodhouse-Sakati syndrome (MIM# 241080) [49], and *CFTR* (rs75389940; category 1; I1234V) which is the most frequently reported cystic fibrosis (MIM# 219700) variant in patients with Arab descent [50], with a corresponding PAR allele frequency of 1.7%. Interestingly, this variant is reported in several Arab populations including in patients from Kuwait, Saudi Arabia, and United Arab Emirates (UAE), highlighting the shared genetic history of populations inhabiting the Gulf region [51].

Out of the 59 potential founder alleles, 20 had at least 1 homozygote carrier in our cohort. These include *RBBP8* (rs373804633; category 3; c.298C>T; p.Arg100Trp), a gene in which bi-allelic variants cause dwarfism and Seckel syndrome (MIM# 606744) [47], *MPL* (rs750046020; category 1; c.317C>T; p.Pro106Leu), which causes a mild (sub-clinical) form of congenital amegakaryocytic thrombocytopenia (MIM# 604498) [52], *CYP1B1* (rs28936700; category 1; c.182G>A; p.Gly61Glu), which causes adult-onset, primary glaucoma type 3 (MIM# 231300) [53] and *MCCC2* (rs150591260; category 1; c.1015G>A; p.Val339Met), known to cause 3-methylcrotonyl CoA carboxylase 2 deficiency (3-MCC deficiency, MIM# 210210) [54]. Given the maximum AF of 3.2% for this *MCCC2* variant in the cohort and the existence of 3 homozygous carriers with no matching clinical symptoms, the pathogenicity of this variant cannot be confirmed as it is common to find homozygous individuals who are asymptomatic due to the phenotypic variability of MCC deficiency. This is supported by expression studies that found a mild reduction in enzymatic activity (only 4–12% activity) in fibroblasts carrying this variant compared to wild type cells [54].

Notably in two genes, two founder alleles were observed in separate subpopulations: *CBS* variants (rs398123151; category 1; c.1006C>T; Arg336Cys in PAR and rs121964972; category 1; c.1058C>T; p.Thr353Met in AFR) and *PADI3* variants (rs139876092; category 2; c.628C>T; p.Arg210Trp in AFR and rs142129409; category 1; c.335T>A; p.Leu112His in SAS) subpopulations. The *CBS* and *PADI3* genes are associated with homocystinuria (MIM# 236200) [55] and uncombable hair syndrome (MIM# 191480) [56] respectively.

### Cumulative gene-level carrier frequency

**Fig. 4:**
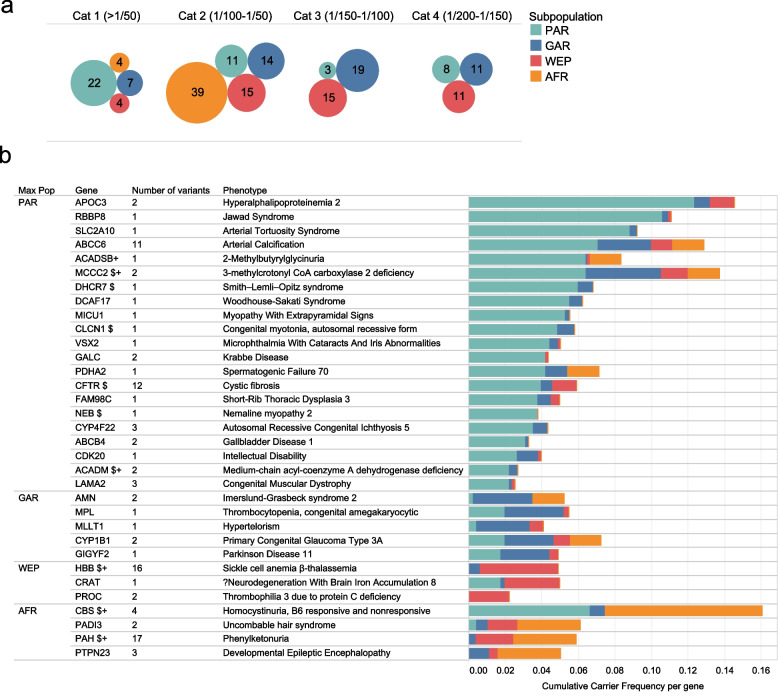
Cumulative gene carrier frequencies based on P/PL variants across various Qatari subpopulations. **a** Number of genes with carrier frequency > 1/200 for the major Qatari subpopulations (SAS is not included due to small sample size). **b** Cumulative gene carrier frequency (GCF) among the major Qatari subpopulations highlighting genes with GCF > 2%. Genes marked with “+” indicate those for which an associated phenotype/biomarker is currently included in the Qatari newborn screening program. Genes marked with “$” refer to genes in the ACMG Tier 3 list

Higher gene carrier frequency (GCF) augments the cumulative risk for recessive diseases, both through consanguinity as well as compound heterozygosity. We examined GCF of P/LP alleles for ARCs among the Qatari subpopulations. We found 150 genes with a GCF > 1/200 in at least one subpopulation (which is the threshold adopted by ACMG to define their Tier 3 gene list) (Additional file 1: Table S6), including 56 genes in category 1 (GCF > 1/50) (Fig. 4a). Comparing the subpopulations, we found a considerable variation in terms of GCF values across various range categories (Fig. 4a). Similarly at gene level, there is variation of GCF values between subpopulations with PAR having the highest GCF burden (Fig. 4b). Of the 150 genes with high CGF (> 1/200), 26 genes are included the ACMG Tier 3 gene panel, and 12 genes are linked to conditions currently tested for in Qatar’s newborn screening program (Additional file 1: Table S6).

### Loss-of-function P/LP variants and human knockouts

Loss-of-function (LoF) alleles were identified in the cohort as having AF < 1% in public datasets and annotated as nonsense, splice-site, and frameshift variants. We identified an average of 52.3 LoF variants per individual genome, 6.5 of which affect our Mendelian panel genes (Additional file 2: Fig. S2a). However, despite the varying levels of consanguinity, no differences were shown in the average number of rare homozygous LoF variants per individual across the QGP ancestries (Additional file 2: Fig. S2b).

In particular, 11 LoF had homozygous carriers (mostly one homozygote per variant) (Additional file 1: Table S7). Three such variants showed phenotypic data for the carriers in support for the known disease phenotype associated with the corresponding genes. First is a nonsense variant (rs76353203; category 3; c.109C>T; p.Arg19*) in *APOC3*, a gene involved in the regulation of blood triglyceride (TG) levels [57], where all carriers had their measured TG levels below the 20th percentile, consistent with a previous report of homozygous carriers having nearly 60% reduction in plasma TG level [47] (variants in this gene causes apolipoprotein C-III deficiency, MIM# 614028) (Additional file 2: Fig. S3a). Second is a splice-site variant in *GJB2* (rs80338940; category 1; c.-23+1G>A), a gene related to autosomal recessive deafness (MIM# 220290) [58] where the only homozygous carrier of the variant in the biobank was reported to be using a hearing aid. This variant is also reported in patients from UAE, Palestine, Egypt, and Algeria [51]. Third is a splice-site variant in *CYP2R1* (rs202011621; category 2; c.118-1G>A) for which the homozygous carrier had an insufficient level of vitamin D (17 ng/ml, reference range > 20 ng/ml), consistent with the fact that variants in this gene cause 25-hydroxyvitamin D deficiency (MIM# 600081) [59], although such levels of vitamin D are not uncommon in the general population.

### Impact of known P/LP alleles on biobank phenotypes

Under a third of the study participants (29.1%) reported parental consanguinity of various degrees (Additional file 2: Fig. S4); therefore, it is expected to identify P/LP variants in ARCs. Of all known-pathogenic variants in our cohort, 22 are being carried in homozygous state (Additional file 1: Table S8). Four P/LP variants had carriers with relevant phenotypes consistent with the known disease phenotype (Table 3). These are missense variant in *CBS* (rs398123151; category 1; c.1006C>T), causing severely elevated homocysteine levels (MIM# 236200) [60] (Additional file 2: Fig. S3b); a missense variant in *ABCG8* (rs137852988; category 1; c.1720G>A; p.Gly574Arg) in a 47-year-old male who self-reported hypercholesterolemia (MIM# 210250) that was consistent with his measured LDL level of 6.3 mmol/L (higher than acceptable range of up to 4.1 mmol/L), despite being on cholesterol-lowering medications and diet management; two variants in two genes linked to eye-related disorders (*CABP4*; rs786205852; category 2; c.81_82insA; p.Pro28fs and *CYP1B1*; rs28936700; category 1; c.182G>A; p.Gly61Glu), in which homozygous carriers reported based on questionnaire having history of macular degeneration (MIM# 610427) [61] and glaucoma (MIM# 231300) [62], respectively. Further two of these four known pathogenic variants (i.e., rs398123151 in *CBS* and rs28936700 in *CYP1B1*) are found to be shared among other Arab nations as reported in the Catalogue for Transmission Genetics in Arabs database (CTGA) [51].

**Table 3:** Carriers of known P/LP variants with biobank phenotypes matching OMIM

dbSNP-id: hgvs_c: hgvs_p	Gene	P/LP category	OMIM phenotypes; OMIM #	Inheritance	Carriers, zygosity	Carriers with relevant biobank phenotype	Biobank phenotype value (normal range)
rs398123151: c.1006C>T: p.Arg336Cys	*CBS*	1	Homocystinuria; MIM# 236200	Recessive	2^a^ HOM	1	High homocysteine levels, 238 (< 15 mmol/L)
rs137852988: c.1720G>A: p.Gly574Arg	*ABCG8*	1	Sitosterolemia 1 involving high LDL-cholesterol, coronary artery disease; MIM# 210250	Recessive	1 HOM	1	High LDL cholesterol level 6.3 (< 4.0 mmol/L)
rs786205852: c.81_82insA: p.Pro28fs	*CABP4*	2	Cone-rod synaptic disorder, congenital nonprogressive; MIM# 610427	Recessive	1 HOM	1	Macular degeneration
rs28936700: c.182G>A: p.Gly61Glu	*CYP1B1*	1	Glaucoma 3 primary congenital type A; MIM# 231300	Recessive	2 HOM	1	Glaucoma
19:11213418 rs771019366 c.269A>G: p.Asp90Gly	*LDLR*	1	Familial hypercholesterolemia type 1; MIM# 143890	Dominant	3 HET	3	High LDL cholesterol levels for the three carriers 9.4, 5.4, and 4.1 (< 4 mmol/L)
15:100230605 rs121918529 c.830C>T: p.Pro277Leu	*MEF2A*	2	Coronary artery disease and myocardial infarction; MIM# 608320	Dominant	4^b^ HET	3	Reported angina +/- family history
3:122003164 rs104893701 c.2393T>G: p.Phe789Cys	*CASR*	2	Hyperparathyroidism, hypocalcemia, hypocalcemia with Bartter; MIM# 601198	Dominant	1 HET	1	High phosphorus, low calcium level on calcium supplementation, hypokalemia and hypomagnesemia
12:114823326 rs104894378 c.710G>C: p.Arg237Pro	*TBX5*	2	HOLT-Oram syndrome; MIM# 142900	Dominant	1 HET	1	proBNP 152.4, underwent cardiac revascularization and angioplasty

^a^One subject has missing value for homocysteine level; however, he is on vitamin B supplementation

^b^One subject did not report chest pain or history of angina but has family history of heart disease from the paternal side

Furthermore, known P/LP variants linked to autosomal dominant conditions (ADCs) were also examined (focusing on those with QGP subpopulation AF < 0.5%), identifying 71 variants in 61 genes (Additional file 1: Table S9). We found four P/LP variants with relevant diseases/phenotypes that support pathogenicity, all of which are missense (Table 3): in *LDLR*, causing familial hypercholesterolemia-1 (MIM# 143890) [63]; *MEF2A*, causing coronary artery disease and myocardial infarction (MIM# 608320) [64]; *CASR*, causing autosomal dominant hypocalcemia (MIM# 601198) [65]; and in *TBX5*, which causes Holt-Oram syndrome (MIM# 142900) [66]. For the latter variant, the diagnosis was not stated in the QBB intake forms of the carrier; however, although not directly related, the variant carrier, despite his young age (34 years), had undergone cardiac revascularization surgery.

### Novel recessive variants associated with extreme quantitative traits

**Fig. 5:**
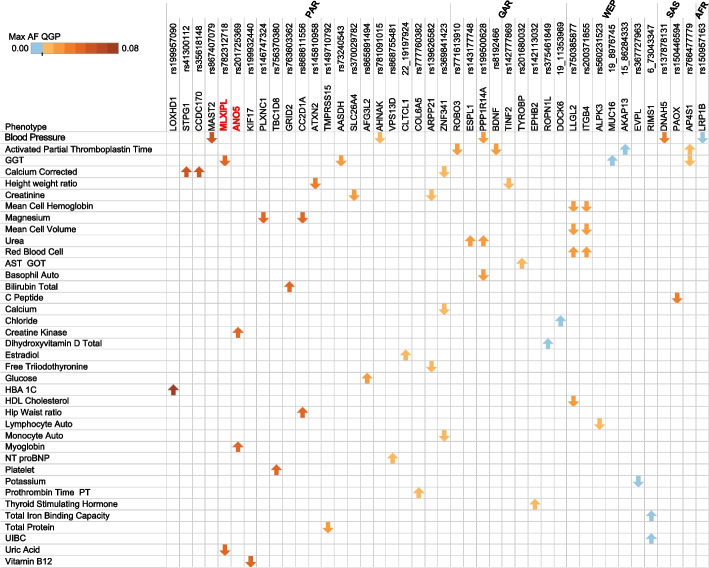
Potential novel pathogenic variants associated with extreme quantitative traits in the study cohort. Shown are 39 variants predicted as being deleterious, for which there are at least two homozygotes and found to be associated with extreme phenotype (at > 95th percentile or < 5th percentile) in 58 quantitative phenotypes in the QBB data. Arrowheads indicate direction of association whether it is > 95th or < 5th percentiles. Highlighted in bold (red color) are two genes with effects supported by the literature as indicated in the main text

Given the burden of consanguinity, the Qatari population is expected to harbor novel pathogenic recessive alleles. Such variants were identified in the current dataset by first selecting Qatari alleles that are rare in the global databases (AF < 1%), have high in-silico prediction sores, and are carried by at least 3 homozygotes. Next, 39 variants from those were found to be associated with extreme biobank phenotypes (Fig. 5, Additional file 1: Table S10). After surveying the literature for these associations, variants in two genes were identified for which there is supporting evidence: *ANO5* (rs201725369; c.172C>T; p.Arg58Trp) and *MLXIPL* (rs782312718; c.2363C>T; p.Thr788Met) (Table 4). Notably, both variants are confined to the PAR subpopulation where their frequency is relatively high (4.6 and 4.7 % respectively) which suggests they are caused by founder effect. *ANO5* variants have been previously linked to several types of myopathies including limb-girdle muscular dystrophy type 2L (LGMD) [67], with symptoms that include severely elevated serum CK [68]. The variant rs201725369 causes a substitution from arginine to tryptophan at position 58 (NM_213599.3) which is predicted to be highly damaging and conserved (CADD, 35; GERP, 5.87; PolyPhen2, 0.99; SFIT, 0). This variant was observed in 5 homozygotes, all of whom had creatine kinase (CK) levels exceeding the 95th percentile of the normal range, and three of them also had elevated levels of myoglobin (median 86.5 ng/ml, which exceeds the reference range max of 72 ng/ml). Notably, CK and myoglobin are known to be correlated biomarkers. As for rs782312718 in *MLXIPL* gene, a cross-sectional study demonstrated an association between this gene and levels of serum uric acid [69]. All 5 homozygotes for this variant have uric acid levels below 5th percentile.

**Table 4:** Potential novel pathogenic variants in the Qatari population with supporting literature

dbSNP-id: hgvs_c: hgvs_p	Gene	Reported literature phenotype	Inheritance	Carriers, zygosity	Carriers with relevant biobank phenotype	Biobank phenotype value (normal range)
rs201725369: c.172C>T: p.Arg58Trp:	*ANO5*	Miyoshi muscular dystrophy 3; MIM# 613319, Muscular dystrophy, limb-girdle, autosomal recessive 12^a^; MIM# 611307	Recessive	5 HOM	5	Creatine kinase level: 563, 572, 823, 1105, and 1811 (29–200 mmol/L)
rs782312718: c.2363C>T: –	*MLXIPL*	Hyperuricemia^b^	Recessive	5 HOM	5	Uric acid level: 183, 172, 163, 159, 196 (155–428 μmol/L)

^a^Referenced in [67, 68]

^b^Referenced in [69]

### Variants in the Qatari population with depleted homozygosity

Selection against deleterious variants in ARCs often results in depletion of homozygotes despite a high carrier frequency. To identify variants with depleted homozygosity in the Qatari population, we examined variants that are rare in the global databases (AF < 1%) and selected those with no observed homozygotes where the number of expected homozygotes is at least 3 assuming random mating. This identified 7 variants, all of which appeared in genes known to cause severe early-onset or life-threatening diseases (Additional file 1: Table S11). Notably, none of these variants was annotated as pathogenic/likely pathogenic in ClinVar, while one was annotated as “DM” in HGMD, a missense variant in *FGG* (rs202132393; c.124G>A: p.Gly42Ser) reported in HGMD to cause congenital afibrinogenemia. The other variants were annotated as having conflicting interpretation or having unknown significance relating to various conditions.

### Rare variant burden analysis

The association between genes with large effect variants and biobank traits was tested using rare variant burden analysis, identifying 13 genes with significant associations (*P* < 1 × 10^−8^) (Table 5), five of which have supporting evidence in the literature (Additional file 2: Fig. S5): (1) association between a putatively damaging variant in *LGI3* (rs149352514; c.1150G>A; p.Gly384Ser) and increased BMI (*P* = 6.6e^−14^); this is consistent with the role of LGI3 as an adipokine with proinflammatory activity that negatively regulates adipogenesis [70]; (2) association between carriers of a *TFR2* variant (rs1002859413; c.554G>A, p.Arg185His) and elevated blood glucose levels (*P* = 8.2e^−9^), consistent with a known link between variants in this gene and hereditary hemochromatosis type III, a condition with complications that result in pancreatic damage and secondary diabetes [71]; (3) association between a splice-site variant (possible loss of function) in *DNAJC15* (rs144620914; c.383-2A>G) and increased levels of the hepatic biomarker ALT-GPT (*P* = 4.2e^−11^), supported by reduced non-alcoholic fatty liver disease (NAFLD), liver steatosis, and fibrosis in *DNAJC15* deficient mice [72]; (4) association between an *RPS6KA3* variant (rs1026040538; c.28C>T; p.Arg10Cys) and sex hormone-binding globulin, which was reported in a GWAS conducted in UK biobank [73]; (5) association between *RGCC* and total cholesterol (c.19C>T; p.Gln7*) consistent with QTL with serum cholesterol being recently identified near *Rgcc* gene in rats [74].

**Table 5:** Significant gene-phenotype associations from rare variant burden analysis

**Gene**	**Variants**	**Biobank phenotype/trait**	**Beta**	**Chi sq**	***P* value**	**Variants**	**RR|RA|AA**^a^	**Supporting evidence from literature**	**Ref**
*DNAJC22*	rs748318571; c.572C>T; p.Ala191Val	Glucose	10.80	69.9	5.95E−17	1	6041|3|1	eQTL in NDDM mice model nearby DNAJC22 homologous gene	-
*HLF*	–; c.465G>T; p.Pro155=	HBA1c	− 10.26	68.2	1.45E−16	1	6041|3|1	-	-
*LGI3*	rs149352514; c.1150G>A; p.Gly384Ser	BMI	16.34	56.1	6.62E−14	1	6037|7|1	LGI3 may be a candidate adipokine that is perturbed in obesity and suppresses adipogenesis through its receptor, ADAM23	70
*ITPK1*	–; c.1111C>T; p.Pro371Ser	ALT-GPT	20.63	50.6	1.12E−12	2	6012|31|2	-	-
	rs567443586; c.1074C>A; p.Ser358Arg								
*MARCH10*	–; c.2026G>C; p.Pro676Ala	Glucose	7.25	44.3	2.70E−11	4	5854|182|9	-	-
	rs116835087; c.1759G>A; p.Gly587Ser								
	rs60472825; c.1285C>T; p.His429Tyr								
	rs529095649; c.113A>G; p.Tyr38Cys								
*DNAJC15*	rs144620914; c.383-2A>G; –	ALT-GPT	43.58	43.5	4.20E−11	1	6039|5|1	Silencing hepatic MCJ attenuates non-alcoholic fatty liver disease (NAFLD) by increasing mitochondrial fatty acid oxidation (reduced NAFLD liver steatosis and fibrosis in MCJ deficient mice)	72
*RPS6KA3*	rs1026040538; c.28C>T; p.Arg10Cys	Sex hormone-binding globulin	130.50	42.1	8.25E−11	1	6040|4|1	Same gene-phenotype association identified in a dataset from UK biobank	73
*FMR1NB*	rs782656279; c.35A>G; p.Asn12Ser	Ferritin	223.39	39.1	3.95E−10	3	6041|1|3	Gene encodes ferritin heavy chain 1 pseudogene 8	-
	rs782029879; c.172C>T; p.Arg58Trp								
	–; c.536A>C; p.Asp179Ala								
*RGCC*	; c.19C>T; p.Gln7^a^	total cholesterol	− 2.57	37.6	8.56E−10	1	6034|10|1	RGD: association in rats with serum cholesterol	74
*STK32C*	rs377335124; c.486C>T; p.Asp175=	BMI	7.78	35.5	2.44E−09	2	6023|20|2	-	-
	rs564621303; c.302-4_302-1dup; –								
*HHAT*	rs757118759; c.62G>C; pArg21Pro	ALT-GPT	26.95	33.8	5.81E−09	2	6032|11|2	-	-
	rs149597734; c.1112C>T; p.Thr371Ile								
*PAX2*	rs199876625; c.453G>A; p.Pro182=	LDL cholesterol	− 2.17	33.2	8.14E−09	1	6027|17|1	-	-
*TFR2*	rs1002859413; c.554G>A; p.Arg185His	Glucose	2.77	33.2	8.22E−09	1	6018|26|1	An association with hereditary hemochromatosis type III, a condition with complications that result in pancreatic damage and secondary diabetes	72

^a^*RR* homozygous reference allele, *RA* heterozygous alternative allele, *AA* homozygous alternative allele

## Discussion

The peculiar structure of Middle Eastern populations illustrated by the Qatari population with its diverse genetic ancestries, runs-of-homozygosity (ROH) [22], and high levels of consanguinity presents a unique opportunity for further exploring the genetics of Mendelian diseases and phenotypes [19]. In these populations, previous studies mainly focused on gene discovery in affected individuals, while burden of disease genetic variation in the general population is yet to be examined. A unique attribute of the Qatar Genome Program (QGP) [28] is the availability of extensive phenotypic data on participants, collected systematically at a single center—the Qatar Biobank [31].

Here, we analyzed 6045 subjects from QGP phase 1 cohort, the largest published so far from the region, leveraging whole genome data and extensive phenotypic information from the Qatar Biobank. We build a comprehensive catalog of known and putatively novel pathogenic variants and their observed impact on biobank traits and use this information to assess the burden in highly consanguineous population, as well as discover novel/founder effect variants and their carrier frequencies, highlighting implications to newborn and pre-marital screening in the region. Notably, we take into consideration the genetic substructure of the population [22] as we correlate known pathogenic variants with phenotypic consequences and conduct enrichment test for rare variants in phenotypic tails and rare variant burden analysis.

Within the highly endogamous Qatari population, 62.5% of the participants were carriers of at least 1 allele of a P/LP variant in genes relating to ARCs, highlighting the increased risk for recessive diseases in the population. On average, each subject carries 0.6 P/LP variant in homozygous status in genes causing ARCs, which is consistent with the general healthy status of the biobank cohort. Notably, we observed distinct differences in the distribution of these homozygotes on a population-level, with 5.8-fold enrichment of homozygotes among Peninsular Arabs who are characterized by longer ROH and have more ancient history relative to other Arab subpopulations [22].

Several known P/LP variants in this study appeared at high frequency in Qatar with some having homozygote genotypes and showing no abnormal biobank traits, suggesting a potential reclassification of variants’ impact to benign, at least in the local population. Other variants appeared at high frequency with confirmed pathogenic phenotype in homozygous state, and these are likely to be founder alleles in specific subpopulation, confirming previously known variants (e.g., in *DCAF17 and CFTR*) and identifying new ones (e.g., in *MPL*, *CYP1B1*, *MCCC2*, *CBS*). This also reflects the underrepresentation of Arab populations in large-scale databases and the value of population-specific reference sets in the identification of pathogenic variants.

ACMG recently updated its recommendation regarding carrier screening for pathogenic variants in populations at risk for recessive disorders to include 97 autosomal recessive genes (Tier 3) [46]. In our cohort, there are more than 50 genes with GCF > 1/50 carriers including 26 present in the ACMG Tier 3 panel; however, only 12 are linked to disorders currently screened for in the Qatari newborn screening program. Therefore, more disorders could be added to the newborn screening panel, particularly those early-onset diseases for which genes have excessively high carrier frequencies (> 1/30), such as *DHCR7*, *CLCN1*, *CFTR*, *NEB*, and *JEP290*. Indeed, this will need to consider the type of subpopulation, given the observed differences, noting there might be an overestimation of GCF in AFR and SAS Arabs due to their low sample size. High consanguinity would be a main driver for the high GCF observed in the cohort, with other cases may be attributable to balancing selection, as illustrated by *HBB* and *CFTR* in which variants are known to confer resistance against Malaria and Cholera, respectively [75, 76].

Consanguineous populations provide an ideal setting for studying LoF variants and human knockouts, uncovering genes that are dispensable or uncritical for fitness [77, 78]. While our data show known P/LP variants are enriched for homozygosity among Peninsular Arabs versus other subpopulations, the distribution of homozygous LoF variants was remarkably similar among various subpopulations (range = 0–5 per person, variance = 0.57). This reflects the cohort’s peculiar population structure and the universal evolutionary constraint of carrying loss-of-function variants in homozygous form, unlike missense variants where subpopulation-differences were seen. In total, 14 participants with homozygote genotype of known P/LP LoF variants were found in our cohort and showed consistent biobank phenotypes. The majority of these belong to the Peninsular Arabs group, including 4 homozygotes with a protein truncating variant in *APOC3*, highlighting the usefulness of this relatively isolated and inbred population in studying gene essentiality.

A number of novel disease-causing alleles in the Qatari population were identified using two approaches. First, variants in *MLXIPL* and *ANO5* were found to cause extreme quantitative traits in hyperuricemia and limb-girdle muscular dystrophy, respectively. Second, rare variant burden analysis was used to identify 5 genes in which rare variants influence quantitative traits relevant to various disease conditions. Candidate pathogenic variants will benefit from replication in larger cohorts as part of the subsequent phases of the Qatar Genome Program as well as functional studies to delineate disease pathophysiology.

## Conclusions

In the era of emerging datasets from under-represented populations [79], consanguineous populations will most likely transform human genetics and may challenge our understanding of Mendelian phenotypes, given most of these are recessively inherited and underlying variant can be naturally observed due to scale of autozygosity. The Qatari biobank cohort advances our knowledge in this context from a Middle Eastern perspective, particularly for populations that share genetic history with the Qatari population as those from Saudi Arabia, UAE, and other Gulf countries [80] as well as the findings among cross-borders Arab tribes that share extended families with presence in several Gulf countries as the Bedouin groups. Of particular interest are the founder pathogenic variants that were identified in the Peninsular Arab (PAR). The current range of phenotypes available at the biobank did not permit the assessment of “subclinical” phenotypic consequences of many variants; nevertheless, as the biobank grows and expands in the near future, larger systematic analyses will be possible leading to the discovery of more variants relevant to population screening programs, eventually lowering/eradicating the associated burden on healthcare systems.

### Supplementary Information

**Additional file 1.** Supplementary Tables S1–S11.

**Additional file 2.** Supplementary Figures S1–S5.

## Data Availability

List of all pathogenic variants reported in this study is provided in the Additional file 1: Table S3. The informed consent given by the study participants does not cover posting of participant level phenotype and genotype data of Qatar Biobank (QBB)/Qatar Genome Project (QGP) in public databases. Access to QBB and QGP raw data can be obtained through submitting a project request at https://www.qatarbiobank.org.qa/research/how-apply and is subject to approval by the QBB IRB committee. As for code availability, the study utilized previously published analysis tools as described in the “ Methods” section.

## Abbreviations

AF: Allele frequency
ACMG: American College of Medical Genetics and Genomics
ARCs: Autosomal recessive conditions
CADD: Combined Annotation Dependent Depletion
GCF: Gene carrier frequencies
GERP: Genomic Evolutionary Rate Profiling
gnomAD: The Genome Aggregation Database
HGMD: The Human Gene Mutation Database
Indels: Insertion-deletions
LoF: Loss of function
P/LP: Pathogenic/likely pathogenic
PCA: Principal component analysis
QBB: Qatar Biobank
QGP: Qatar Genome Program
ADM: Admixed Arabs
AFR: African Arabs
GAR: General Arabs
PAR: Peninsular Arabs
SAS: South Asians Arabs
WEP: West Eurasians and Persian Arabs
SNVs: Single-Nucleotide Variants
