# Supplementary material for: Burden of Mendelian disorders in a large Middle Eastern biobank
Source: Genome Med. 2024 Apr 8;16:46. doi: 10.1186/s13073-024-01307-6 (PMC11000384; doi:10.1186/s13073-024-01307-6)
Supplement: Supplementary file 2 — Additional file 2. Supplementary Figures S1–S5. [file 13073_2024_1307_MOESM2_ESM.docx]

**Supplemental Figures**

**Fig. S1: Per-individual** **count of** **pathogenic (P/LP) variants in the QGP subpopulations**

P/LP variants per individual shown for each QGP subpopulation.

**Fig. S2**: **Distribution of LoF variants in the Qatari cohort**

To the left is the distribution of rare LoF vriants in the QGP subpopulations, both, in genome wide and Mendelian panel genes, while to the right is the distribution of rare homozygous LoF variants in the QGP subpopulations, both, in the whole genome and Mendelian panel genes.

**Fig. S3. (a)** Distribution of triglyceride amongst carriers of rs80338940 (*APOC3*) and **(b)** distribution of homocysteine amongst carriers of rs398123151 (*CBS*)

**Fig. S4: Parent consanguinity among Qatar Biobank participants**

Self-reported parent consanguinity among 6,045 participants from the Qatar biobank used in the current study.

**Fig. S5: Highlighted significant hits from rare-variant burden analysis in the biobank cohort**

Rare-variant burden analysis was done using 6,045 Qatari genomes and 58 quantitative phenotypes from the Qatar Biobank. REF, HET, and HOM, refers to reference, heterozygote, and homozygote genotypes, respectively. Number of carriers for each of the genotypes is shown on top of each boxplot.
